# Individualized Treatment Approach for Rectal Adenocarcinoma in the Setting of Congenital Neutropenia

**DOI:** 10.7759/cureus.56383

**Published:** 2024-03-18

**Authors:** Nicole W Forneris, Solly Chedid

**Affiliations:** 1 Medicine, William Carey University College of Osteopathic Medicine, Hattiesburg, USA; 2 Oncology, Singing River Hospital System, Gulfport, USA

**Keywords:** distal rectal cancer, adult neutropenia, personalized medicine (pm), neupogen, colorectal oncology, individualized treatment, nodal metastasis, rectal adenocarcinoma, locally advanced rectal cancer, severe congenital neutropenia (scn)

## Abstract

Congenial neutropenia is a rare genetic disorder that puts individuals at risk of life-threatening bacterial infections early in life, and the current standard of care includes the use of colony-stimulating factors or curative intent bone marrow transplant. Cancer treatment strategies that include surgery, chemotherapy, radiation, and immunotherapy present significant challenges to an individual with a baseline immunodeficiency as seen in this condition. Evidence-based national guidelines aid physicians and patients in moving through complex cancer care regimens. However, these are altered when the intensity of the patient's comorbidities puts them at increased risk of developing a potentially life-threatening infection. Here, we present a patient treated for rectal carcinoma in the setting of severe congenital neutropenia.

## Introduction

The characterization of neutropenia can be complex as the level of neutrophils naturally fluctuates and even varies seasonally [1]. Congenial neutropenia is a rare genetic disorder of bone marrow failure that has historically caused early death from bacterial infections. While neutropenia is described as absolute neutrophil count (ANC) count less than 1.5 K/uL, this can be further described as mild with an ANC of 1.0-1.5 K/uL, moderate with an ANC of 0.5-1.0 K/uL, severe with an ANC of 0.2-0.5 K/uL, and very severe with an ANC of less than 0.2 K/uL [1]. The only curative treatment for congenital neutropenia would be a stem cell transplant. However, many barriers to treatment exist, and these patients are treated chronically with colony-stimulating factors.

Approaching treatment of a newly diagnosed malignancy in an individual whose neutrophil count is blunted can be particularly problem-some, even when the condition is somewhat stabilized by colony-stimulating factors, close monitoring, and intermittent antibiotics for acute infections. Cytotoxic chemotherapy treatment regimens are typically accompanied by a profound negative impact on the rapidly dividing cells within the bone marrow, which includes neutrophils. Neutropenic fever is a common occurrence that can result in treatment delays, poor disease outcomes, and death [2]. Colony-stimulating factors are often used in cancer patients without baseline blunted neutrophil counts to prevent treatment delays, particularly in more aggressive cytotoxic treatment regimens. Without careful monitoring of of the clinical and hematologic state of the cancer patient with congenital neutropenia, in addition to ongoing use of scheduled growth factors throughout treatment, and early infection response, individuals with congenital neutropenia would be at risk for a severe life-threatening infection. Furthermore, newer cancer treatments, including immunotherapy, would present challenges in a patient with congenital neutropenia as it requires a competent immune system in order to be effective. Undergoing surgery or even port placement in anticipation of treatment can also provide an easy opening for infection. Evidence-based guidelines, like those provided by the National Comprehensive Cancer Network (NCCN), provide evidence-based treatment options and an instrumental approach to cancer treatment, guiding physicians and patients along the complex, ever-expanding understanding of both tumor biology and treatment [3,4]. However, physicians need to weigh the risks and benefits of adhering to a strict treatment approach and take into account significant comorbidities. Here we demonstrate a case of rectal adenocarcinoma with an individualized treatment approach in the setting of the rare, heterogenous disease of severe congenital neutropenia.

## Case presentation

The patient is a 42-year-old caucasian male smokeless tobacco user with a history of epilepsy, anemia, and congenital neutropenia on Filgrastim. His chronic, severe neutropenia was diagnosed at age nine years old and had resulted in two hospital admissions for infection in the year leading up to his presentation. His family history included lung cancer in his maternal grandmother and unspecified cancer in his maternal grandfather. He presented with obstipation, severe abdominal pain, and scrotal pain. He denied melena, hematochezia, or fevers.

**Figure 1:**
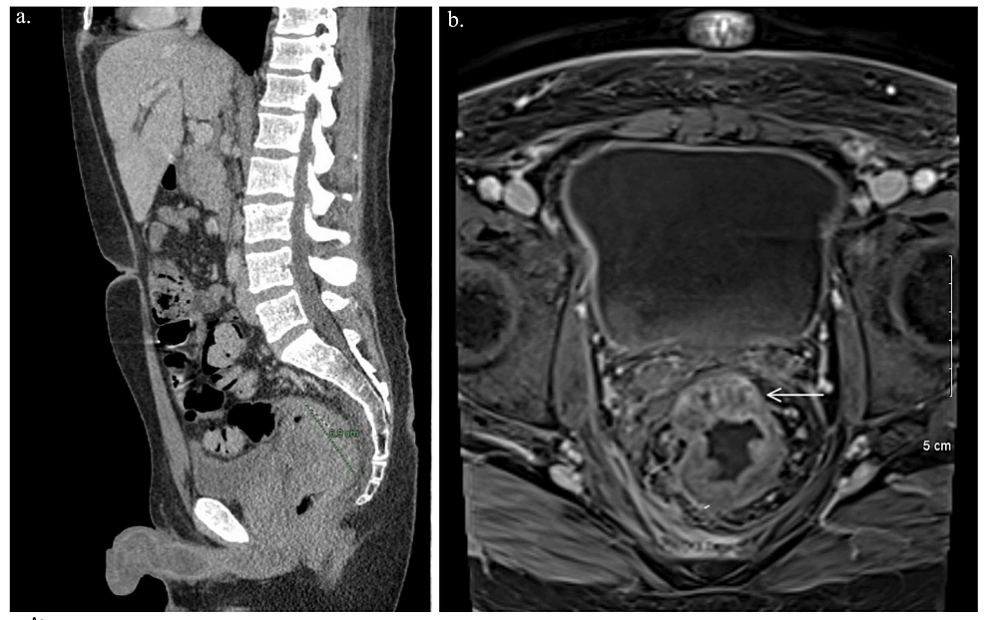
Imaging study results (a) CT abdomen pelvis scan showing constipation with short-segment irregular heterogenous wall thickening of the rectosigmoid colon measuring 6.9 cm (in software notation with dotted line); (b) MRI of the pelvis showed stage cT3b N2a disease with suspicious extra mesorectal lymph node and a defect in the right lateral wall of the rectum, suspicious for contained perforation.

Workup included Computed Tomography (CT) imaging of the abdomen and pelvis with contrast showed constipation with short-segment irregular, heterogeneous wall thickening of the rectosigmoid colon measuring 7.0 cm, right nephrolithiasis and hepatosplenomegaly (Figure 1a). Colonoscopy the next day showed a large rectal mass extending to the anal verge. Biopsy pathology confirmed adenocarcinoma with intact Mismatch Repair protein (MMR protein) expression and low probability of Microsatellite Instability (MSI). Clinical staging was performed with a Magnetic Resonance Imaging (MRI) scan of the pelvis showed stage cT3b N2a disease with a suspicious extra mesorectal lymph node and a defect in the right lateral wall of the rectum, suspicious for contained perforation (Figure 1b).

**Figure 2:**
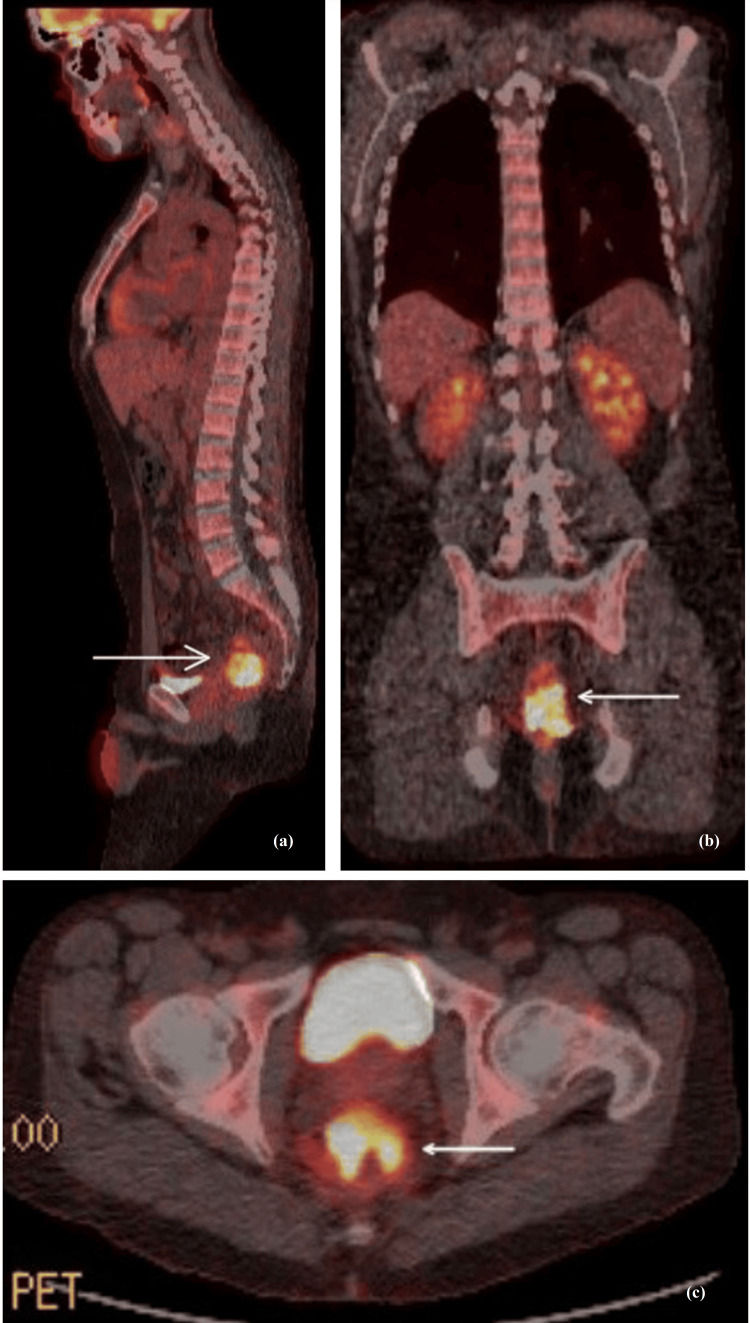
PET-CT scan images Positron Emission Tomography-Computed Tomography imaging (PET-CT) scan images show a primary neoplastic rectal mass with SUV of 16.5 and multiple centimeters and subcentimeter presacral lymph nodes without associated FDG uptake. (a) Sagittal view (b) Coronal view (c) Axial view

Positron Emission Tomography-Computed Tomography imaging (PET-CT) scan showed a primary neoplastic rectal mass with Semiquantative Measurement of Uptake in Tissue (also noted SUV) of 16.5 and multiple centimeters and subcentimeter presacral lymph nodes without associated Flurodeoxyglucose (FDG) uptake (Figure 2). He was leukopenic at 2.8 K/uL (ANC of 0.25 K/uL), anemic with hemoglobin of 7.2 g/dL with elevated ferritin of 568 ng/mL and normal platelets of 244 x 10^9^/L. MyRisk genetic testing showed no clinically significant mutations identified.

In light of his chronic severe congenital neutropenia, the case was discussed at a local tumor board meeting with a plan to use a Peripherally Inserted Central Catheter (PICC) line rather than the typical Mediport as it would be easier to replace in the setting of infection. Additionally, it was decided to have a short course induction radiation therapy followed by resection and adjuvant chemotherapy with folinic acid, fluorouracil, and oxaliplatin (FOLFOX); oxaliplatin 85 mg/m^2 ^IV 14D:1, leucovorin 400 mg/m^2^ IV infused over 2 hours 14D:1, 5-fluorouracil 400 mg/m^2^ IV Bolus infused over two hours 14D:1, and 5-flurouracil 2,400 mg/m^2^ IV infusion pump started D1 and administered over 46 hours.

He received 4,500 cGy of radiation therapy to the pelvis and then underwent total mesorectal excision with colo-anal anastomosis. Full pathologic staging was performed following resection. He was staged IIIB (ypT3, pN1a, pMX) with pathology consistent with mucinous rectal adenocarcinoma, 5.3 cm in greatest dimension, notably invading through the muscularis propria into the pericolonic or perirectal tissue. He had one positive lymph node out of 74 total sampled lymph nodes. His surgical margins were clear to 0.2 cm and he had no evidence of lymphovascular invasion (LVI).

**Figure 3:**
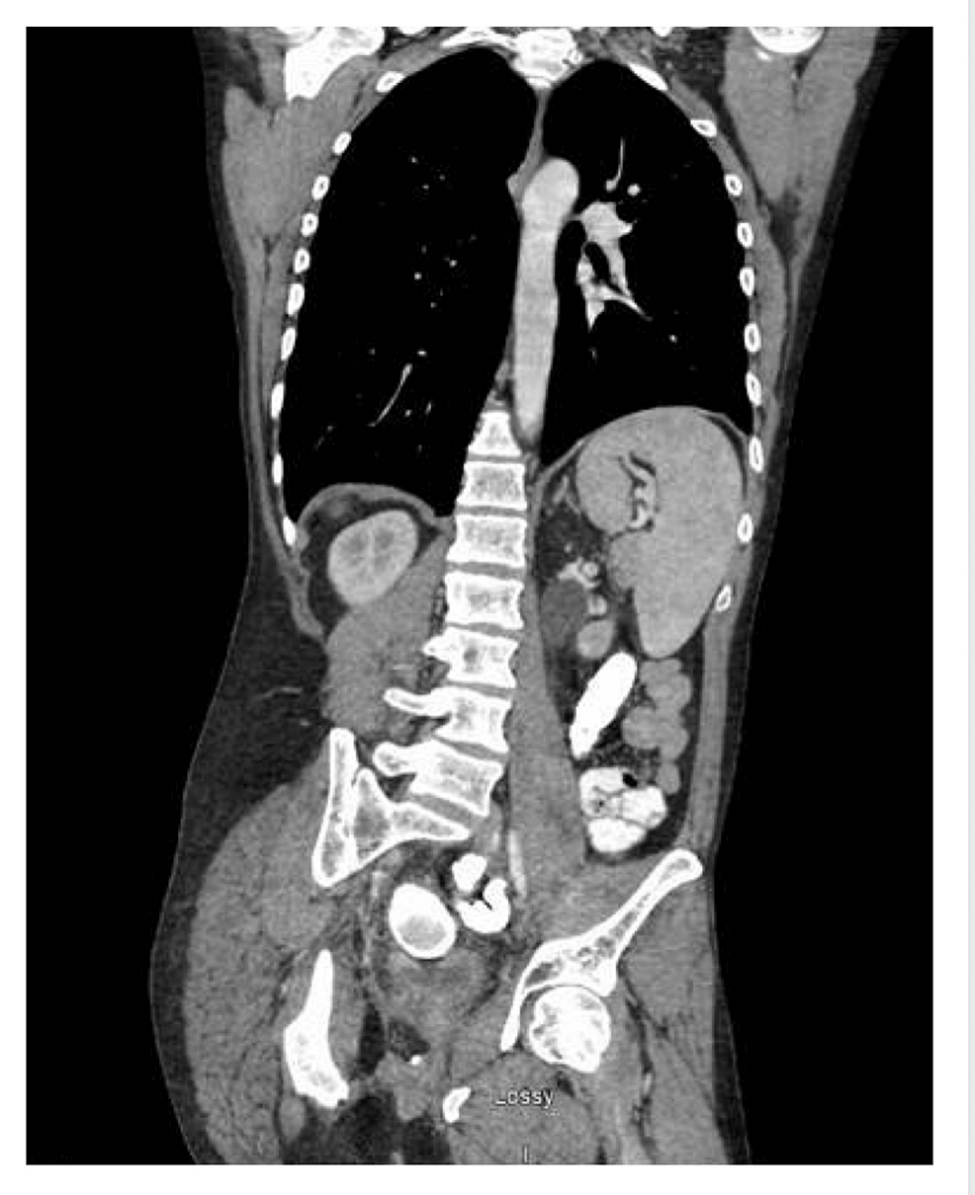
CT abdomen pelvis showing post-surgical changes and no evidence of recurrent disease Coronal view

He ultimately received eight cycles of the aforementioned adjuvant FOLFOX regimen without dose reductions, however cycle 3 was delayed two weeks due to terrorist ransomware against the hospital, and cycle 5 was delayed two weeks due to myelosupression. The patient was already receiving filgrastim in the care of his severe congenital neutropenia and continued this throughout treatment. Post-treatment CT abdomen pelvis showed no evidence of the disease (Figure 3) and the patient underwent a successful colostomy reversal.

## Discussion

Severe congenital neutropenia is diagnosed early in life and associated with recurrent, frequent life-threatening infections. Standard of care for severe congenital neutropenia includes maintenance treatment of ANC between 1-2 K/uL with Granulocyte-Colony Stimulating Factor (G-CSF) agents like filgrastim or curative-intent treatment with stem cell transplant [5]. There is a risk of transformation to Myelodysplastic Syndrome (MDS) and Acute Myeloid Leukemia (AML). Thus, regular, yearly bone marrow biopsies are recommended for surveillance [6]. This patient’s surveillance bone marrow biopsies prior to his diagnosis of rectal cancer and during treatment, fortunately, did not indicate transformation to MDS or AML. Unfortunately, for many patients, there are many barriers to transplant [7] and ongoing, chronic management of diseases like congenital neutropenia may be the only option. This patient was able to complete care for his rectal cancer despite some financial barriers to care.

As severe congenital neutropenia is a rare condition, the literature on severe congenital neutropenia and its association with concordant malignancy is sparse and largely focused on the risk of transformation to hematologic malignancies without reference to established cases or guidance for management of malignant solid tumors [8]. Furthermore, a comorbid condition like severe congenital neutropenia would exclude a patient from entry into malignant solid tumor clinical trials. Management of a case like this with a new, malignant solid tumor diagnosis in the setting of severe congenital neutropenia has not been described. In the above case report, we have described a patient with T3 N1 MX staged rectal adenocarcinoma. Recommendations for treatment of rectal cancer of this stage in a patient without congenital neutropenia would be expected to vary based on the patient’s performance status and other co-morbidities. Such recommendations would include neoadjuvant chemotherapy (with a FOLFOX regimen or, alternatively, a CAPEOX regimen using capecitabine and oxaliplatin). Additionally, neoadjuvant long-course chemoradiation with capecitabine or 5-fluorouracil versus short-course chemoradiation followed by surgical resection would be considered. The benefit of short-course radiation as part of treatment has been examined retrospectively in a single-site study, but the application of such to a general population requires further examination [9]. Also, dependent on pathologic staging and disease features, possible additional adjuvant systemic therapy may include immunotherapy for advanced disease. For this patient, each step of potential treatment options was examined carefully with the goal of minimizing toxicity from the combination of treatment and the underlying congenital immunodeficiency. Even the decision of how to safely administer chemotherapy was discussed with surgery and interventional radiology at a tumor board meeting. This discussion included the risks and benefits of placing a PICC line, central line, or the traditional Mediport. It was felt that a closely monitored PICC line would allow for easy removal if needed in addition to rapid response infection management. Proceeding with a regimen of short course radiation, surgery, and adjuvant chemotherapy was deemed safest for the patient with strict instructions and close monitoring and management for signs of infection.

The patient did well under these recommendations and had only two episodes concerning infection during treatment with prompt, positive response to antibiotics. His treatment delays, as described above were relatively minimal treatment delays and not due to his underlying immunodeficiency. This has resulted in the resolution of his disease on post-treatment imaging and colostomy takedown.

## Conclusions

While rare, severe congenital neutropenia is considered a significant comorbidity that has significant impacts on a patient’s treatment plan following a cancer diagnosis. We presented an individualized approach to treatment planning for a patient with rectal cancer in the setting of severe congenital neutropenia. The patient has done well overall to date as a result of this individualized treatment protocol with favorable post-treatment imaging and colostomy takedown.
